# Subperichondrial Hematoma of Alar Cartilage: A Case Report

**DOI:** 10.22038/IJORL.2023.71166.3417

**Published:** 2023-07

**Authors:** Mehmet Ali Say

**Affiliations:** *Department of Otolaryngology, Cerkezköy State Hospital, Tekirdağ, Turkey.*

**Keywords:** Alar Cartilage, Nasal trauma, Subperichondrial Hematoma

## Abstract

**Introduction:**

Cartilage deformation may develop due to congenital and trauma-related hematomas. Early diagnosis and treatment are imperative to prevent aesthetic and functional complications related to alar cartilage hematomas.

**Case Report:**

An 8-year-old male presented with a major alar cartilage hematoma with a nasal fracture as a result of trauma. The patient underwent surgery on the 1st day of trauma for alar cartilage hematoma drainage and nasal bone reduction. No functional or cosmetic complications were observed in the patient's postoperative 1st month.

**Conclusion:**

Subperichondral hematomas of the alar cartilage are rarely observed after nasal trauma and early diagnosis and treatment are important to prevent possible complications.

## Introduction

The nose is one of the most frequently traumatized parts of the body; therefore, the nasal bone is the most often fractured in maxillofacial trauma (1,2). 

Soft tissue and nasal cartilage injuries are usual in patients with nasal fractures. Trauma-related hematomas are common in the nasal septum region; however, trauma-related hematomas are rare in the alar cartilage (3-4). Cartilage deformations may develop because of congenital and trauma-related hematomas (5).

Early diagnosis and treatment are essential to prevent aesthetic and functional complications related to alar cartilage hematomas. In this article, we report a rare case of alar cartilage hematoma with nasal fracture resulting from trauma in an 8-year-old male patient.

## Case Report

An 8-year-old boy presented to the outpatient clinic with nasal pain and swelling 1 day after a fall. The patient was conscious, oriented, and cooperative with others. 

**Fig1:**
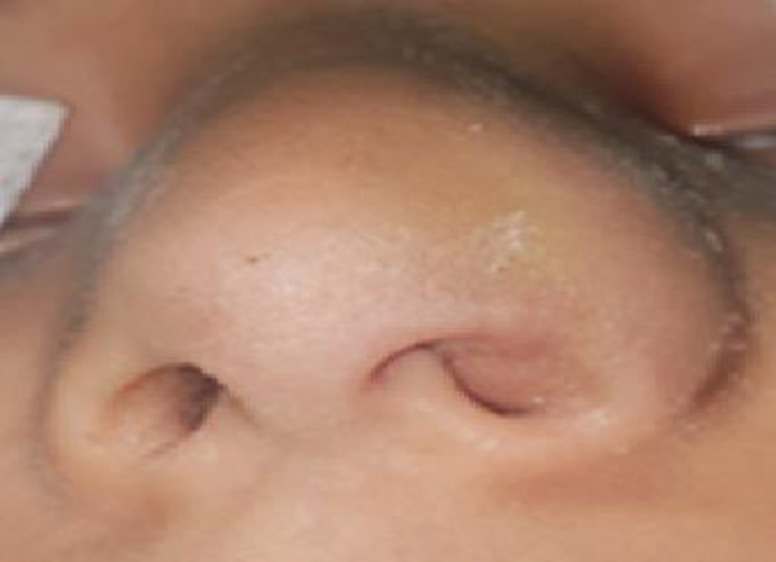
Intraoperative image of alar cartilage hematoma

Swelling was observed in the left alar region of the patient's nose. No septal hematoma was detected on anterior rhinoscopy. No septal hematoma was detected on anterior rhinoscopy. Swelling and hematoma were observed in the patient’s left alar cartilage. (Figure 1).

**Fig 2:**
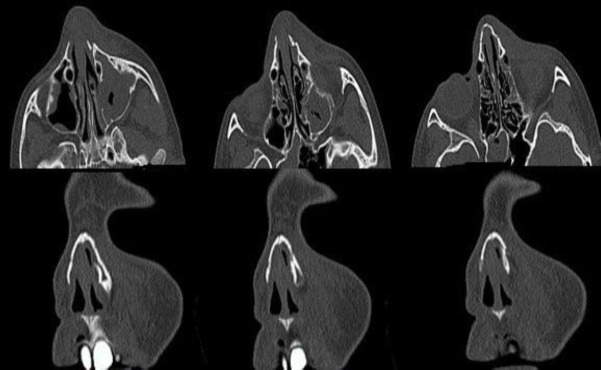
Preoperative maxillofacial CT image of the patient

Maxillofacial tomography revealed a fractured line on the left side of the nasal bone (Figure 2).

**Fig 3:**
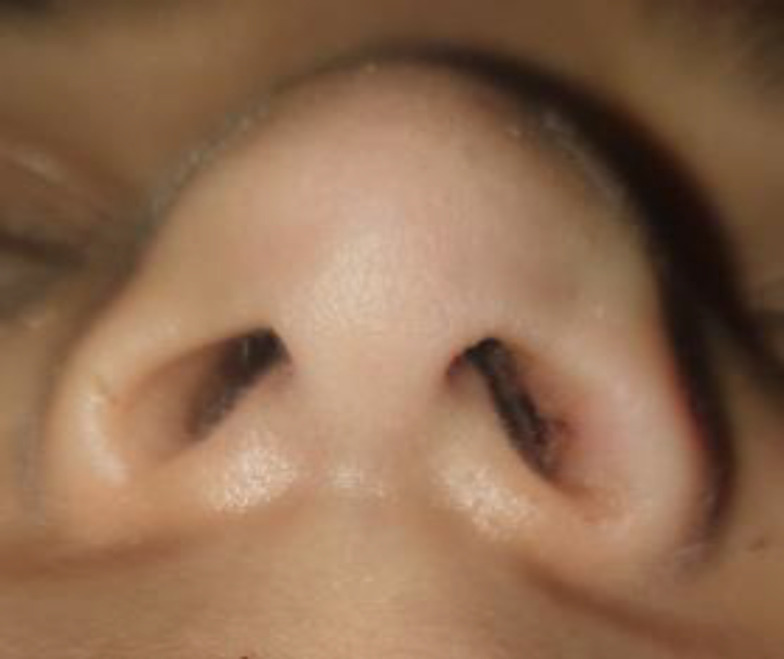
Image of the patient at the postoperative follow-up

The patient underwent surgery on the 1st day of trauma for alar cartilage hematoma drainage and nasal bone reduction. During the operation, an incision was made 2-3 mm above the left infracartilaginous incision. An alar cartilage hematoma was drained, and nasal reduction was performed. A tamponade (Merocel, Medtronic Inc., Minneapolis, MN, USA) was placed in the left nasal cavity to keep the incision site open. Amoxicillin/clavulanic acid prophylaxis was administered. The tamponade was removed on the 3rd postoperative day. The patient was followed up for 6 months in terms of functional and cosmetic complications. No functional or cosmetic complications were observed in the patient's postoperative 6st month. (Figure 3) Written consent was obtained from the patient for this study.

## Discussion

The alar cartilage forms the external appearance of the nose and determines its position of the nose type and size. The alar cartilage also contributes to respiratory function. The alar cartilage has a C-shaped structure and plays a role in nasal entrances and nostrils formation (6). Bicycle accidents, sports injuries, intentional injuries, and home accidents can lead to nasal fractures in children (7).

 Nasal fractures may be accompanied by hematomas, which can cause abscess formation and cartilage necrosis (8). Although septal hematoma is the most common nasal hematoma caused by trauma, very few subperichondrial hematoma cases of lateral cartilage have been reported in the literature (4,9,10). The predisposing factor for alar cartilage hematoma is nasal trauma, observed primarily in pediatric patients (4). Septal and alar hematomas are more commonly seen in the pediatric population because the cartilage tissue has a weak and loosely adherent mucoperichondrium; consequently, most alar hematoma cases in the literature are based on pediatric patients (11.^12^).

Cartilage tissue with an avascular structure acquires oxygen from the perichondrium capillaries. Because of the accumulation of hematoma between the perichondrium and cartilage, the alar cartilage cannot receive sufficient glucose and oxygen. Deformity and loss of function occur in the alar cartilage owing to tissue necrosis and resorption (13), resulting in serious side effects, such as cosmetic deformation and nasal breathing deterioration (4,9,13). Patients should be followed-up for at least six months to monitor cosmetic complications. Alar cartilage hematomas are difficult to diagnose owing to their low incidence, which may result in delayed treatment (3,14). In alar cartilage hematoma, as in septal hematoma, treatment involves urgent surgical and antimicrobial therapy because of the deformities that appear when treatment is delayed (4). Our patient underwent hematoma evacuation on the first day after trauma, and no functional or cosmetic complications were observed during the follow-up.

## Conclusion

Subperichondral hematomas of the alar cartilage are rarely observed after nasal trauma and early diagnosis and treatment of these conditions are important to prevent possible complications.
